# Understanding social care need through primary care big data: a rapid scoping review

**DOI:** 10.3399/BJGPO.2022.0016

**Published:** 2022-09-21

**Authors:** Glenn Simpson, Lucy Mutindi Kaluvu, Jonathan Stokes, Paul Roderick, Adriane Chapman, Ralph Kwame Akyea, Francesco Zaccardi, Miriam Santer, Andrew Farmer, Hajira Dambha-Miller

**Affiliations:** 1 Primary Care Research Centre, University of Southampton, Southampton, UK; 2 Faculty of Health, Social Care and Medicine, Edge Hill University, Ormskirk, UK; 3 Centre for Primary Care and Health Services Research, University of Manchester, Manchester, UK; 4 Department of Public Health, University of Southampton, Southampton, UK; 5 Electronics and Computer Science, University of Southampton, Southampton, UK; 6 Leicester Real World Evidence Unit, Leicester Diabetes Centre, University of Leicester, Leicester, UK; 7 Nuffield Department of Primary Care Health Sciences, University of Oxford, Oxford, UK

**Keywords:** social care, social care need, social support, primary health care, big data

## Abstract

**Background:**

A more comprehensive understanding and measurement of adult social care need could contribute to efforts to develop more effective, holistic personalised care, particularly for those with multiple long-term conditions (MLTC). Progress in this area faces the challenge of a lack of clarity in the literature relating to how social care need is assessed and coded within variables included in primary care databases.

**Aim:**

To explore how social care need is assessed and coded within variables included in primary care databases.

**Design & setting:**

An exploratory rapid scoping review of peer-reviewed articles and grey literature.

**Method:**

Articles were screened and extracted onto a charting sheet and findings were summarised descriptively. Articles were included if published in English and related to primary and social care using data from national primary care databases.

**Results:**

The search yielded 4010 articles. Twenty-seven were included. Six articles used the term ‘social care need’, although related terminology was identified including ‘need factors’, ‘social support’, and ‘social care support’. Articles mainly focused on specific components of social care need, including levels of social care usage or service utilisation and costs incurred to social care, primary care, and other providers in addressing needs. A limited range of database variables were found measuring social care need.

**Conclusion:**

Further research is needed on how social care need has been defined in a UK context and captured in primary care big databases. There is potential scope to broaden the definition of social care need, which captures social service needs and wider social needs.

## How this fits in

Relatively few studies have examined social care need, despite the availability of longitudinal records collating millions of variables on both health and social care. Understanding which variables are available to capture social care need, how these are recorded in practice, and standardising of coding could be useful in progressing research in this area. A more comprehensive understanding and measurement of adult social care need could potentially contribute to efforts to develop more effective, holistic personalised care, particularly for those with MLTC, who often have complex needs requiring a range of joined-up health and social care services, as well as wider social interventions.

## Introduction

The burden of managing long-term conditions remains an overarching challenge for healthcare systems in the UK and worldwide.^1,2^ An unprecedented rise in life expectancy coupled with changes in lifestyle has resulted in an increase in the prevalence of chronic conditions such as cardiovascular diseases, hypertension, depression, and rheumatoid arthritis.^2^ This has seen the number of individuals developing ≥2 long-term chronic conditions rise substantially. MLTC is commonly associated with ageing, with more than two-thirds of adults aged ≥65 years having ≥2 long-term chronic conditions.^3,4^ A rising MLTC burden is also evident among those aged ≤45 years who reside in socioeconomically deprived areas.^1^ People with MLTC face numerous challenges, such as functional decline, higher disability, poorer mental health, as well as an overall reduced quality of life.^5^ Studies show that people with MLTC have the highest level of health service use and healthcare expenditure. For example, care and support provided for MLTC account for >50% of all primary and secondary care costs,^6^ primarily as a result of high levels of health service utilisation, in particular GP appointments, emergency service use, admissions, and medication use.^2,7,8^ In response, this has necessitated service integration to more holistically address the needs of people with multiple conditions.^8^ Compared with individuals with a single condition, those with MLTC often require a combination of both health and social care services.^9^

Both conceptually and in practice, it can be difficult to clearly distinguish between healthcare need and social care need. This review used the national framework for NHS continuing health care and NHS-funded nursing care^10^ to define and differentiate social care need and healthcare need. Healthcare need is defined in the framework as *‘the treatment, control, management or prevention of a disease, illness, injury or disability, and the care or aftercare of a person with these needs (whether or not the tasks involved have to be carried out by a health professional)’*. Although there is not a formal legal definition of social care need in the UK, it can be described as *‘one that is focused on providing assistance with activities of daily living, maintaining independence, social interaction, enabling the individual to play a fuller part in society, protecting them in vulnerable situations, helping them to manage complex relationships and (in some circumstances) accessing a care home or other supported accommodation’*.^11^ Importantly, this definition of social care need encompasses both a person’s social care provision needs and their wider social needs, which enable full participation in society.

Compared with healthcare need in people with MLTC,^3,7,8^ research evidence on social care need is more limited, largely as a result of a lack of clarity both in the literature and in practice of how to define and measure social care need. Despite the availability of longitudinal records collating millions of variables on both health and social care, there is uncertainty in the literature on standardisation of variables to capture ‘social care need’ and its related coding systems. Understanding which variables are available to capture social care need, how these are recorded in practice, and standardising of coding could be useful in progressing research in this area. Accordingly, an exploratory rapid scoping review was conducted to identify and gain a broad overview of the key research evidence relating to how social care need is assessed and coded within variables included in primary care databases.

## Method

### Review approach

The study followed the Preferred Reporting Items for Systematic reviews and Meta-Analyses extension for Scoping Reviews (PRISMA-ScR) guidelines for scoping reviews.^12^ The scoping review method was used as it allowed for a rapid mapping of key existing evidence and sources available in an emerging field of research.^13^ Analytically, the scoping review primarily focused on the variables that are used to capture social care need, how these are recorded in practice, and standardised in coding.

### Search strategy

Systematic electronic searches were conducted from inception to 26 October 2021 on the following electronic databases: MEDLINE, Embase, the Cochrane Library, Web of Science, the Cumulative Index to Nursing and Allied Health Literature (CINAHL), Trip Database, Social Care Online, and Social Sciences Citation Index. For searches of electronic databases, free-text and MeSH terms were used and limited to ‘primary care’ and ‘social care’. Details of search terms are available in Table 1. Manual searching of bibliographies were also conducted. The views of topic experts were also sought to identify additional sources.

**Table 1.** Search terms results table

MeSH and free-text search terms	Databases searched	Filters or refined by	Sources identified, *n*
((primary care or (Primary Health Care or Primary care provider or primary care facility or Primary Care Physicians)).mp. or (Primary Health Care/ or "Continuity of Patient Care"/ or Patient-Centred Care/ or Progressive Patient Care/)) and Social care.mp.	MEDLINE	All dates searched.Language: restricted to English or English Language.	801
((primary care or primary health) and social care).mp.	Embase	All dates searched.Language: restricted to English.	821
'primary care' and ’social care' (free text AND MeSH terms)	Cochrane Library	All dates searched.Language: restricted to English.	224
[ "primary care" OR "primary healthcare"] AND [“social care” OR “adult social care"] AND [Location: “united kingdom"' including this term only]	Trip Database	All dates searched.Language: restricted to English.	12
(primary care or primary health care or primary healthcare or general practice or GP) AND (social care or adult social care)	Cumulative Index to Nursing and Allied Health Literature(CINAHL)	All dates searched.Language: restricted to English.Geographic subset and journals: UK and Ireland.	458
'primary care' and ’social care' (free text AND MeSH terms)	Social Care Online	All dates searched.Language: restricted to English	355
[Primary care OR primary healthcare (Topic) and social care (Topic)	Web of Science (includes search of Social Sciences Citation Index database)	All dates searched.Language: restricted to English.Countries or regions: UK Publications.	1335
Hand searches	N/A	N/A	4
Total			4010

N/A = not applicable.

### Inclusion and exclusion criteria

Articles were eligible for inclusion if published in the English language, were geographically located in the UK, and were focused on primary and social care. Quality assessment criteria are not a priority for scoping reviews,^13^ therefore extracted articles were not excluded on this basis.

### Study selection and data extraction

All articles identified were imported into the Rayyan collaborative review platform for screening, which was conducted in blinding mode. Rayyan enabled rapid screening of retrieved sources. Titles and abstracts were screened, with each article assessed for relevance according to the inclusion criteria. Full-text articles were retrieved. Both screening and data extraction were conducted independently by two reviewers and disagreement resolved by discussion. A data-charting form was used to collate the studies and identify key characteristics (see Supplementary Appendix S1). Reviewers extracted the article reference and date, the stated aim of the study, results, and variables or indicators that had been used to capture ‘social care need’. Any disagreement between reviewers about study or variable inclusion was resolved through discussion until a consensus was reached.

### Summarising and analysis

The data gathered by the review were iteratively synthesised descriptively, through the use of counts to summarise article characteristics (that set out the number, type, and quality of studies extracted and collated social care need variables identified), the data-charting technique, and interpretation of the findings by sifting and sorting material.^14^ The main social care need variables identified were collated and are presented in Supplementary Appendix S1.

## Results

### Screening, inclusion, and exclusion of studies

A total of 4010 articles were found by the review. After title and abstract screening using the Rayyan review tool, 61 articles were eligible for full-text screening. Following full-text screening, a further 34 articles were excluded, for reasons including:

- did not produce findings or discuss the scoping review’s aim of how social care need is assessed and coded within variables included in primary care databases;
- the geographical focus was not on the UK; and
- the reviewers were unable to retrieve the full-text version of a small number of articles in the time available for the scoping review owing to publisher access permissions or paywalls.

**Figure 1.**
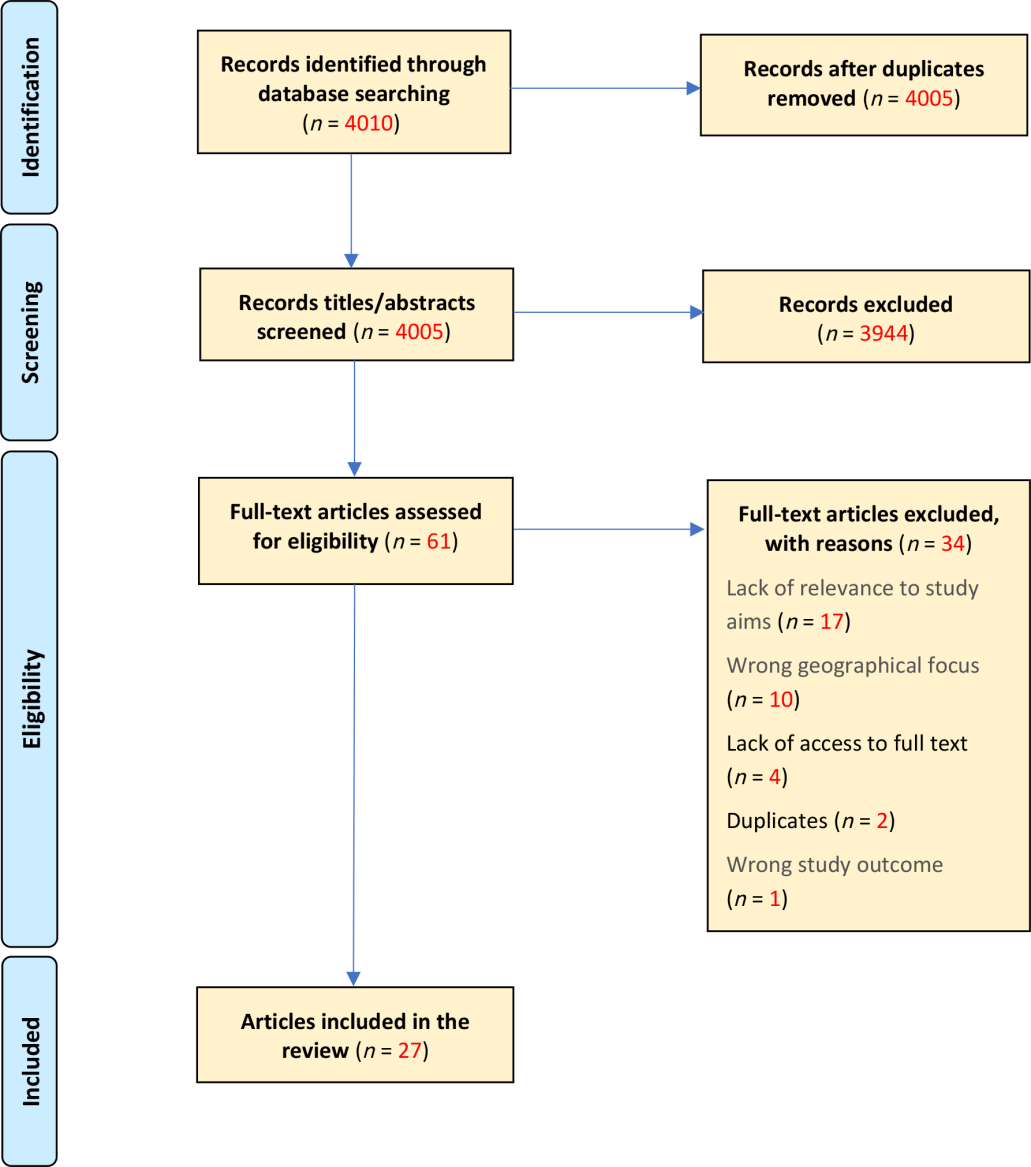
Adapted PRISMA flow chart. Explaining the study’s documentary screening and inclusion and exclusion process.

A total of 27 final articles were included in the review.^15–41^ The flowchart of the screening process used for the review, including reasons for exclusion, are summarised in Figure 1.

### Characteristics of included studies

Most studies were located in the UK (*n* = 24),^15–32,34–39^ with a small number of UK and international studies identified (*n* = 3).^33,40,41^ Included articles were published between 2000 and 2021. The main study settings identified were either primary and/or secondary care, social care, or both. The main population groups examined in the studies were people with health conditions (patients) (*n* = 9),^21,24,29,33,34,37–41,41^ people in age categories ≥55 years (*n* = 5),^18,19,28,37,40^ service users (*n* = 3),^23,27,32^ and staff or healthcare professionals (*n* = 3).^22,27,33^ The most frequent sources of data used by studies were secondary data (*n* = 16),^15–35,37–39,41,31,35,37–39,41^ which is pooled data from primary sources, and primary data (*n* = 9).^22–26,27,29,32,33,36,36^

The main study designs used were cross-sectional (*n* = 5),^16,30,33,38,40^ mixed methods (*n* = 5),^22,23,27,32,33^ cohort study (*n* = 4),^15,18,24,36^ longitudinal (*n* = 3),^22,29,33^ systematic reviews (*n* = 2),^21,35^ and policy or evidence reviews (*n* = 2).^25,41^ A small number of studies were specifically based on datasets extracted from UK primary care databases including Clinical Practice Research Datalink (*n* = 2)^16,35^ and Secure Anonymised Information Linkage Databank (*n* = 2).^17,18^

#### Social care need(s)

Six studies specifically referred to the term ‘social care need’ although no standardised definition of social care need was identified.^19,21,25–33,33^ There were three studies using terms related to social care need, including ‘need factors’ (*n* = 1),^28^ ‘social support’ (*n* = 1),^29^ and ‘social care support’ (*n* = 1).^23^ Fifteen studies examined specific aspects of social care need, measuring the level of social care use or service utilisation (*n* = 8)^15,19,22,23,28,33,34,36^ and the costs of and the expenditure incurred to social care, primary care, and other care sectors or providers in addressing social care need (*n* = 7).^24,27,29–32,39^

The review found a relatively limited sample of studies containing data variables or coding lists from UK primary care database studies.^15–18^ These are shown in Supplementary Appendix S1. One study^19^ set out indicators measuring five aspects of ‘social care need’: *‘visual or hearing impairment, social isolation, access to an unpaid carer, activities of daily living/functional markers, other health problems recorded in social care data’.* Another investigated the *‘recording of social factors in linked electronic health records of individuals aged ≥65 years, to assess the potential of these data to identify the social determinants of disease burden and uptake of healthcare interventions’*.^16^ This study used a number of indicators to measure ‘social factors’, including *‘religion, ethnicity, immigration status, small area-level deprivation, place of residence (including communal establishments such as care homes), marital status and living arrangements (e.g. living alone, cohabitation)’*.^16^ Reeve and Baker,^20^ albeit in the context of population health need, used a number of social care need-related indicators (described as ‘indices of social care provision’), which were focused on older people: *‘gross SSA* [Social Security Administration] *expenditure on elderly people per capita 75 years and over; people supported in residential/nursing care; the number of home help/care contact hours; and households receiving intense home care’*.

The social care-related quality of life (SCRQoL) framework was used by some studies,^21,22^ which is based on the eight outcome domains of social care-related quality of life set out in the Adult Social Care Outcomes Toolkit. This was employed as an indicator of the quality of life of social care service users and to measure the outcomes and benefits of service provision, and specific interventions.^23,24^

Other indicators or measures utilised as surrogates of social care need included: ‘activities of daily living’ or ‘functional markers’ of need (for example, personal care, walking, bathing, or dressing);^19^ indicators measuring service utilisation (for example, home care, day care, and meals usage based on the number of days per year); using adult social care assessment to identify and measure demand for services; and, access to or presence of an unpaid or informal carer.^19,25,26^ One study^27^ that evaluated reablement outcomes used a questionnaire to ask service users if they felt they had *‘enough money to live on’*, which acted as a ‘proxy indicator’ of their perceived financial situation in the context of understanding the impacts of wider social determinants of health.

## Discussion

### Summary

This rapid scoping review was conducted to identify and gain a broad overview^13^ of the key research evidence relating to how social care need is assessed and coded within variables included in primary care big databases. The study also sought to identify the range and type of ‘social care need’ variables or indicators used in the existing research literature. A common definition of social care need was not evident across included studies. Indeed, there was limited evidence of the use of the specific term ‘social care need’ across the literature. Other related terminology was used to describe aspects of social care need in specific population cohorts and in the context of service provision such as ‘need factors’,^28^ ‘social support’,^29^ and ‘social care support’.^23^ This review identified studies with a narrow analytical focus on particular variables or aspects of care need. For example, some studies considered and framed care need in terms of the costs incurred to social care, primary care, and other care providers, and/or in relation to levels of social care usage or utilisation.^15,19,20,28,30,31^ A few studies^20,25,27,32^ specifically focused on need identified through statutory social care service provision or interventions (for example, reablement or home help). While others^21,24,33^ examined what could be described as wider socioeconomic need or social determinants affecting health and wellbeing (for example, employment status, deprivation or social gradient, personal or household financial situation, and barriers to accessing services).

### Strengths and limitations

This is the first review, to the authors’ knowledge, that collates evidence on how social care need has been examined in UK primary care big database studies. This review permitted rapid collation of evidence across a number of databases, with the inclusion of manual searches and expert input. Search terms were purposely broad, aimed at a comprehensive search and overview of research in this field.

However, the broad search criteria may have omitted studies in more specific subject fields or subdisciplines. As the review aims were only focused on UK databases, it was appropriate that searches were limited to UK studies and those published in the English language, although this will have resulted in the omission of relevant international evidence. Robust double peer-review and data extraction were carried out, but as this is a scoping review a formal quality appraisal process of included works was not conducted. It is important to acknowledge that unlike systematic reviews, the scoping review method does not attempt to achieve comprehensive coverage of the available research evidence, rather *‘to map the concepts underpinning a research area and the main sources and types of evidence available’*.^13^ Therefore, more research is needed to identify additional sources and relevant evidence to validate the findings of this scoping review and further understand this research topic more generally.

### Comparison with existing literature

This scoping review highlighted the limited literature focused specifically on social care need and associated indicators or variables, particularly in relation to those found in primary care big databases. This limited evidence base makes it difficult to systematically compare the findings of this scoping review with the findings of previous studies.

The review indicates a lack of universal agreement in the literature on which definition to use when describing social care needs for adults, a finding replicated in other work.^42^ It confirms the findings of other research such as that suggesting activities of daily living (ADL) is frequently employed as a proxy indicator of social care need,^43^ and it is also a useful measure for distinguishing the degree and type of care needs.^20^ This measure, which is based on local authority assessment of social care need data, does not capture the full range of social care and wider social needs of individuals.^42,43^ As Dunatchik and colleagues^43^ remark, an ADL *‘based approach also has the limitation that it is very task-focused and may underplay the importance of other aspects of life such as social contact and maintaining a sense of purpose, which are outcomes under the Care Act 2014’*. While the review found evidence of other measures associated with social care need, these were mainly ‘service indicators’ measuring the type of social care provision and levels of service utilisation, and ‘indicators of costs’ incurred to care providers,^43^ which are even narrower measures that fail to capture the full range of social care need identified in the Care Act 2014.

### Implications for research and practice

The findings of this review highlight the need for further research on how social care need has been defined in a UK context and captured in primary care big databases. In relation to policy and practice, there is scope to broaden the definition of social care need, especially beyond ADL measures, and develop more comprehensive measures or indicators of social care need, capturing both the full range of social care service provision needs and wider social needs (including social determinants of health) experienced by individuals. A more comprehensive understanding and measurement of adult social care need could potentially contribute to efforts to develop more effective, holistic personalised care, particularly for those with MLTC, who have complex and diverse needs requiring a range of joined-up healthcare and social care services, as well as wider social interventions.

## Availability of data and materials:

Data used during the current study are available from the corresponding author on reasonable request.

## Data

Data used during the current study are available from the corresponding author on reasonable request.

## References

[1] Barnett K, Mercer SW, Norbury M (2012). Epidemiology of multimorbidity and implications for health care, research, and medical education: a cross-sectional study. Lancet.

[2] Cassell A, Edwards D, Harshfield A (2018). The epidemiology of multimorbidity in primary care: a retrospective cohort study. Br J Gen Pract.

[3] Nguyen H, Chua K-C, Dregan A (2020). Factors associated with multimorbidity patterns in older adults in England: findings from the English Longitudinal Study of Aging (ELSA). J Aging Health.

[4] Kingston A, Robinson L, Booth H (2018). Projections of multi-morbidity in the older population in England to 2035: estimates from the Population Ageing and Care Simulation (PACSim) model. Age Ageing.

[5] Dambha-Miller H, Simpson G, Hobson L (2021). Integrating primary care and social services for older adults with multimorbidity: a qualitative study. Br J Gen Pract.

[6] Stokes J, Guthrie B, Mercer SW (2021). Multimorbidity combinations, costs of hospital care and potentially preventable emergency admissions in England: a cohort study. PLOS Med.

[7] Sheringham J, Asaria M, Barratt H (2017). Are some areas more equal than others? Socioeconomic inequality in potentially avoidable emergency hospital admissions within English local authority areas. J Health Serv Res Policy.

[8] Marszalek K, Parry W, Jayatunga W, Deeny S (2019). A descriptive analysis of health care use by high cost, high need patients in England. https://www.health.org.uk/publications/a-descriptive-analysis-of-health-care-use-by-high-cost-high-need-patients-in-england.

[9] Violán C, Foguet-Boreu Q, Roso-Llorach A (2014). Burden of multimorbidity, socioeconomic status and use of health services across stages of life in urban areas: a cross-sectional study. BMC Public Health.

[10] Department of Health and Social Care (2022). National framework for NHS continuing healthcare and NHS-funded nursing care. https://www.gov.uk/government/publications/national-framework-for-nhs-continuing-healthcare-and-nhs-funded-nursing-care.

[11] Jepson A (2020). Adult social care and support in Scotland. https://sp-bpr-en-prod-cdnep.azureedge.net/published/2020/12/3/92a1d806-219e-11ea-b692-000d3a23af40/SB20-78.pdf.

[12] Tricco AC, Lillie E, Zarin W (2018). PRISMA extension for scoping reviews (PRISMA-ScR): checklist and explanation. Ann Intern Med.

[13] Tricco AC, Lillie E, Zarin W (2016). A scoping review on the conduct and reporting of scoping reviews. BMC Med Res Methodol.

[14] Dambha-Miller H, Simpson G, Hobson L (2021). Integrated primary care and social services for older adults with multimorbidity in England: a scoping review. BMC Geriatr.

[15] Stafford M, Deeny SR, Dreyer K, Shand J (2021). Multiple long-term conditions within households and use of health and social care: a retrospective cohort study. BJGP Open.

[16] Jain A, van Hoek AJ, Walker JL (2017). Identifying social factors amongst older individuals in linked electronic health records: an assessment in a population based study. PLoS One.

[17] Lyons RA, Jones KH, John G (2009). The SAIL databank: linking multiple health and social care datasets. BMC Med Inform Decis Mak.

[18] Hollinghurst J, Fry R, Akbari A (2019). External validation of the electronic Frailty Index using the population of Wales within the Secure Anonymised Information Linkage Databank. Age Ageing.

[19] Bardsley M, Billings J, Dixon J (2011). Predicting who will use intensive social care: case finding tools based on linked health and social care data. Age Ageing.

[20] Reeves D, Baker D (2004). Investigating relationships between health need, primary care and social care using routine statistics. Health Place.

[21] Dickson K, Sutcliffe K, Rees R, Thomas J (2017). Gaps in the evidence on improving social care outcomes: findings from a meta-review of systematic reviews. Health Soc Care Community.

[22] Gridley K, Aspinal F, Parker G (2019). Specialist nursing support for unpaid carers of people with dementia: a mixed-methods feasibility study. Health Serv Deliv Res.

[23] Caiels J, Forder J, Malley J (2010). Measuring the outcomes of low-level services: final report. https://www.pssru.ac.uk/pub/dp2699.pdf.

[24] Panagioti M, Reeves D, Meacock R (2018). Is telephone health coaching a useful population health strategy for supporting older people with multimorbidity? An evaluation of reach, effectiveness and cost-effectiveness using a “trial within a cohort.”. BMC Med.

[25] Bolton J, Provenzano P (2017). Six steps to managing demand in adult social care: a performance management approach — full report. https://ipc.brookes.ac.uk/publications/six-steps-to-managing-demand-in-adult-social-care-a-performance-management-approach-full-report.

[26] Ford JA, Kharicha K, Clarke CS (2017). Service use of older people who participate in primary care health promotion: a latent class analysis. BMC Health Serv Res.

[27] Beresford B, Mann R, Parker G (2019). Reablement services for people at risk of needing social care: the more mixed-methods evaluation. Health Serv Deliv Res.

[28] Forder J, Gousia K, Saloniki E-C (2019). The impact of long-term care on primary care doctor consultations for people over 75 years. Eur J Health Econ.

[29] Wheelwright S, Permyakova NV, Calman L (2020). Does quality of life return to pre-treatment levels five years after curative intent surgery for colorectal cancer? Evidence from the ColoRectal Wellbeing (CREW) study. PLoS One.

[30] Jayatunga W, Asaria M, Belloni A (2019). Social gradients in health and social care costs: analysis of linked electronic health records in Kent, UK. Public Health.

[31] Shand J, Morris S, Gomes M (2021). Understanding health and care expenditure by setting — who matters to whom?. J Health Serv Res Policy.

[32] Callaghan L, Towers A-M (2014). Feeling in control: comparing older people’s experiences in different care settings. Ageing Soc.

[33] Bunn F, Burn A-M, Goodman C (2016). Comorbidity and dementia: a mixed-method study on improving health care for people with dementia (codem). Health Serv Deliv Res.

[34] Liu D, Pace ML, Goddard M (2021). Investigating the relationship between social care supply and healthcare utilization by older people in england. Health Econ.

[35] Oyinlola JO, Campbell J, Kousoulis AA (2016). Is real world evidence influencing practice? A systematic review of CPRD research in NICE guidances. BMC Health Serv Res.

[36] Sampson EL, Candy B, Davis S (2018). Living and dying with advanced dementia: A prospective cohort study of symptoms, service use and care at the end of life. Palliat Med.

[37] Iliffe S, Wilcock J (2017). The UK experience of promoting dementia recognition and management in primary care. Z Gerontol Geriatr.

[38] Raine R, Lewis L, Sensky T (2000). Patient determinants of mental health interventions in primary care. Br J Gen Pract.

[39] Franklin M, Berdunov V, Edmans J (2014). Identifying patient-level health and social care costs for older adults discharged from acute medical units in england. Age Ageing.

[40] Oviedo-Briones M, Laso ÁR, Carnicero JA (2021). A comparison of frailty assessment instruments in different clinical and social care settings: the frailtools project. J Am Med Dir Assoc.

[41] Allen K, Glasby J (2013). “The billion dollar question”: embedding prevention in older people’s services--ten “high-impact” changes. British Journal of Social Work.

[42] Raymond A, Bazeer R, Barclay C (2021). Our ageing population: how ageing affects health and care need in England. https://www.health.org.uk/publications/our-ageing-population.

[43] Dunatchik A, Icardi R, Blake M (2019). Predicting unmet need for social care. Journal of Long-Term Care.

